# Beyond cholesterol: regression of carotid plaques in an adolescent-a case report

**DOI:** 10.1093/ehjcr/ytag603

**Published:** 2026-08-18

**Authors:** Chao-Chun Zou, Meng-Qin Pan, Min Wang

**Affiliations:** Department of Endocrinology, Children’s Hospital, Zhejiang University School of Medicine, National Clinical Research Center for Children and Adolescents’ Health and Diseases, No. 3333 Binsheng Road, Hangzhou 310052, China; Department of Family Medicine, Zhangjiajie People's Hospital, No.192 Guyong Road, Zhangjiajie, Hunan Province 427000, China; Department of Endocrinology, Children’s Hospital, Zhejiang University School of Medicine, National Clinical Research Center for Children and Adolescents’ Health and Diseases, No. 3333 Binsheng Road, Hangzhou 310052, China

**Keywords:** Atherosclerosis, Hypercholesterolaemia, Ultrasound, Treatment, Case report

## Abstract

**Background:**

Sitosterolaemia is a rare lipid disorder characterized by massive accumulation of phytosterols. Due to its non-specific clinical features and the lack of routine sterol quantification, diagnostic delays are common, leading to inappropriate dietary interventions that may paradoxically accelerate vascular damage.

**Case summary:**

We herein describe the case of a 13-year-old boy with sitosterolaemia and early-onset atherosclerosis against a background of a 7-year diagnostic delay. Following the identification of a paradoxical pro-atherogenic effect of a standard lipid-lowering diet, treatment with a plant-oil-restricted diet and cholestyramine resulted in a 51.7% reduction in carotid plaque thickness.

**Discussion:**

This case highlights the necessity of screening for sitosterolaemia in paediatric patients with unexplained carotid plaque. Plaque resolution was achieved only after replacing conventional lipid-lowering strategies with targeted phytosterol-lowering interventions and specialized dietary modifications.

Learning points

- Sitosterolaemia should be a primary differential diagnosis in paediatric patients with refractory xanthomas or early-onset atherosclerosis.
- Further research is needed to establish standardized phytosterol-lowering thresholds and to evaluate the long-term impact of selective sterol absorption inhibitors.

## Introduction

Phytosterols—including sitosterol, stigmasterol, and campesterol—are plant-derived sterols.^1,2^ Dietary intake of phytosterols may reduce coronary heart disease (CHD) risk.^3^ However, sitosterolaemia (phytosterolaemia), first described in 1974,^4^ is a rare autosomal recessive disorder caused by mutations in ABCG5 (Type 2 sitosterolaemia)or ABCG8 (Type 1 sitosterolaemia),^5^ leading to markedly elevated blood phytosterol levels (5–200 times normal). The disease manifests in diverse forms, with xanthomas as the most typical feature. Other features include corneal arcus, arthralgia, hepatosplenomegaly, haemolytic anaemia, thrombocytopenia, and early-onset atherosclerotic CHD.^6^ Misdiagnosis is common due to limited awareness and nonspecific symptoms.

## Summary figure

**Figure:**
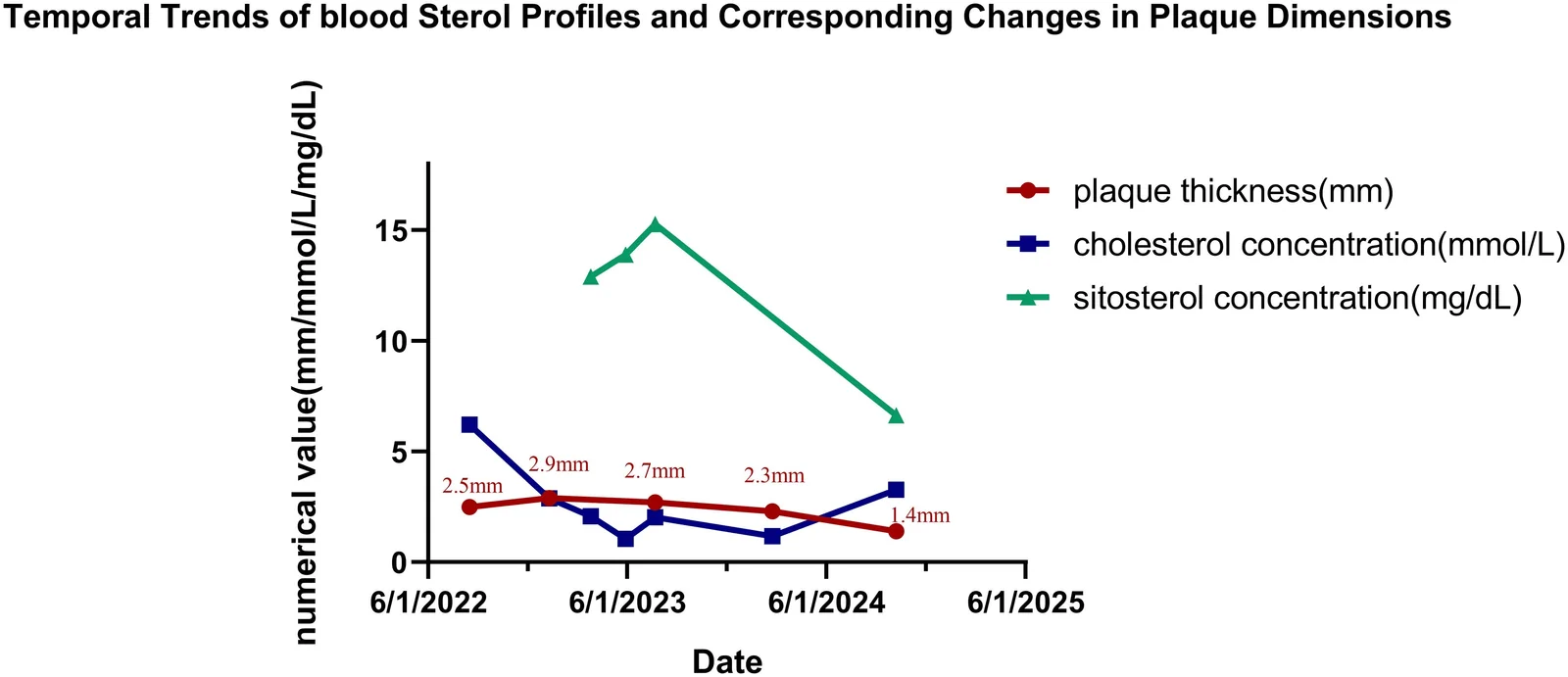


## Case presentation

**Figure 1:**
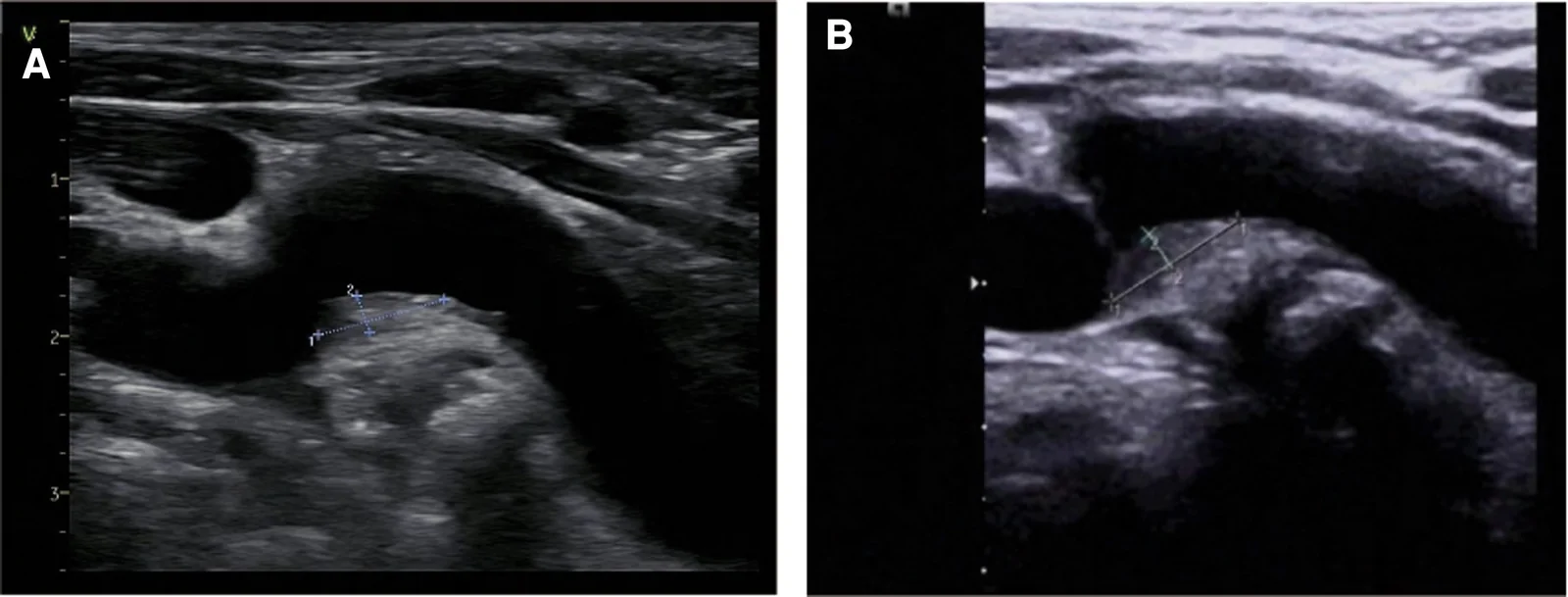
Vascular ultrasound. (*A*) There was an 8.3 × 2.5 mm atheromatous plaque at the right innominate artery bifurcation. (*B*) A persistent 10.0 × 2.9 mm atheromatous plaque was observed at the same site 5 months later. The cross indicates the atheromatous plaque.

The patient’s clinical history spanned a 7-year diagnostic odyssey beginning at age 6, with multiple misdiagnoses including benign fibrous histiocytoma (biopsy-confirmed in 2021) (see Supplementary material online, *Figure S1*) and progressive pseudorheumatoid dysplasia (MRI-suggested in 2022) (see Supplementary material online, *Figure S2*), accompanied by recurrent joint pain and enlarging xanthomas over the elbows, patellae, and Achilles tendons. There was no history of fever, arthritis, or movement disorders. Laboratory investigations—including C-reactive protein, electrolytes, ferritin, erythrocyte sedimentation rate (23 mm/h), antinuclear antibody, rheumatoid factor, anti-streptococcal haemolysin O, HLA-B27, and tuberculin test—were all normal or negative. A second biopsy of buttock papules in August 2022 finally confirmed xanthomatosis (see Supplementary material online, *Figure S1*), leading to a presumptive diagnosis of familial hypercholesterolemia (FH) prior to referral, which was confirmed by LDL-C of 6.22 mmol/L. Given the presence of xanthomas and hypercholesterolemia, carotid duplex ultrasound was performed for cardiovascular risk assessment, which incidentally revealed an 8.3 × 2.5 mm atheromatous plaque at the right innominate artery bifurcation (*Figure 1A*). After 5 months of ezetimibe and dietary restriction, LDL-C decreased to 2.88 mmol/L (111 mg/dL), yet follow-up imaging showed paradoxical plaque progression to 10.0 × 2.9 mm (*Figure 1B*). This atypical trajectory—advancing atherosclerosis despite therapeutic LDL-C—prompted multidisciplinary re-evaluation. He had no history of hypertension or neurological symptoms.

Based on the findings of skin and tendon xanthomas, hypercholesterolemia, and no family history of FH, sitosterolaemia was strongly suspected. Blood sterol quantification confirmed profound hyperphytosterolaemia, with a sitosterol level of 12.91 mg/dL (normal range: <1 mg/dL), stigmasterol of 0.72 mg/dL, and campesterol of 7.84 mg/dL. Whole-exome sequencing identified compound heterozygous mutations in the *ABCG5* gene: a paternal pathogenic splicing mutation (c.1464–1G > C) and a maternal likely pathogenic missense mutation (c.1418C > G), confirming a diagnosis of Type 2 sitosterolaemia. Haematological analysis revealed an increased proportion of stomatocytes (49.4%), while electrocardiogram and echocardiography were unremarkable.

Family screening identified heterozygous carriers in both parents and the sister; all were asymptomatic, with mildly elevated campesterol in the mother and sister (1.13 and 1.17 mg/dL, respectively; reference <0.8 mg/dL) but normal in the father, and a negative family history for premature coronary artery disease or hypercholesterolemia.

**Figure 2:**
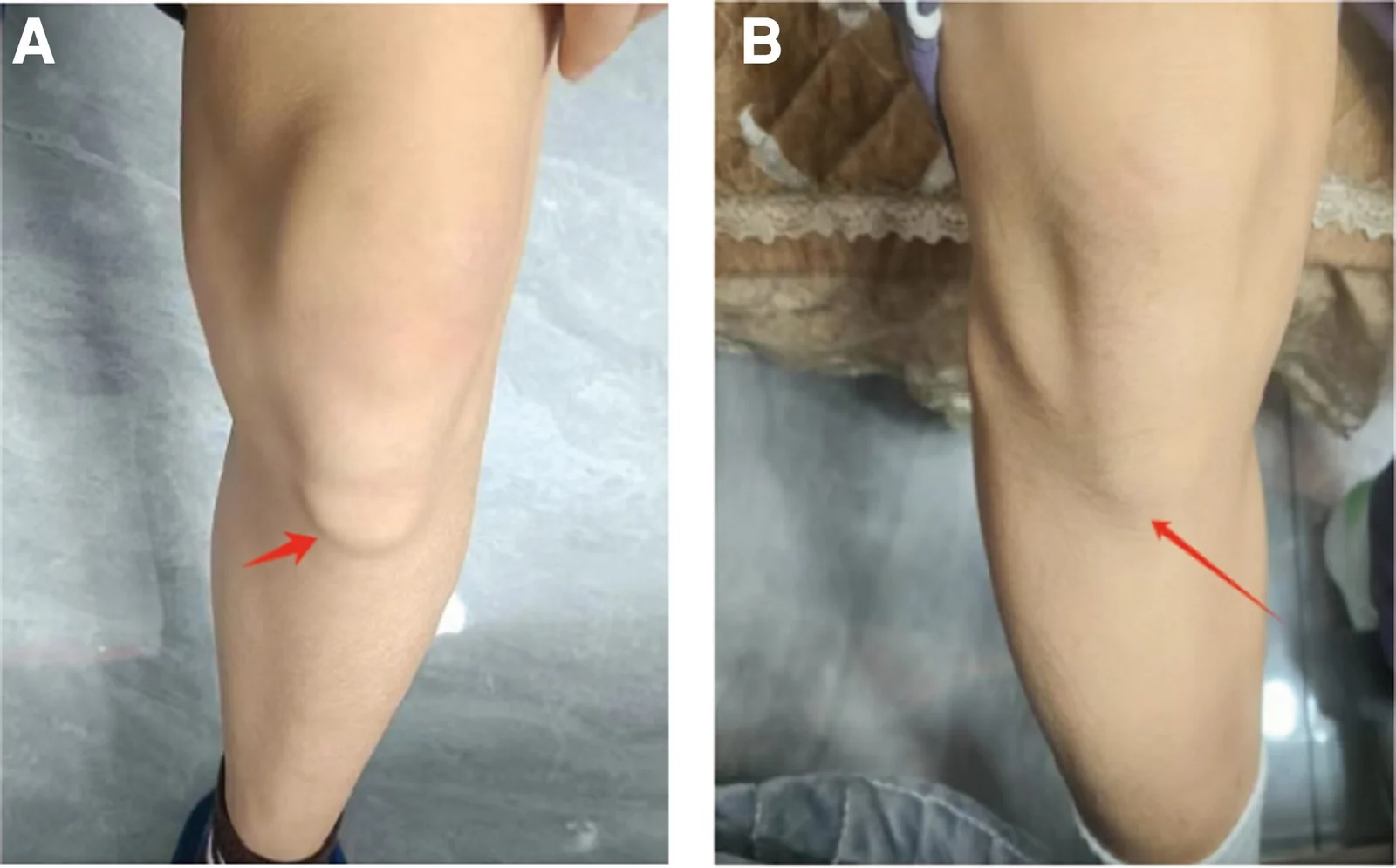
Xanthoma changes. (*A*) Xanthoma in the patellar tendon before intervention ( arrow); (*B*) xanthoma in the patellar tendon after intervention (red arrow).

**Figure 3:**
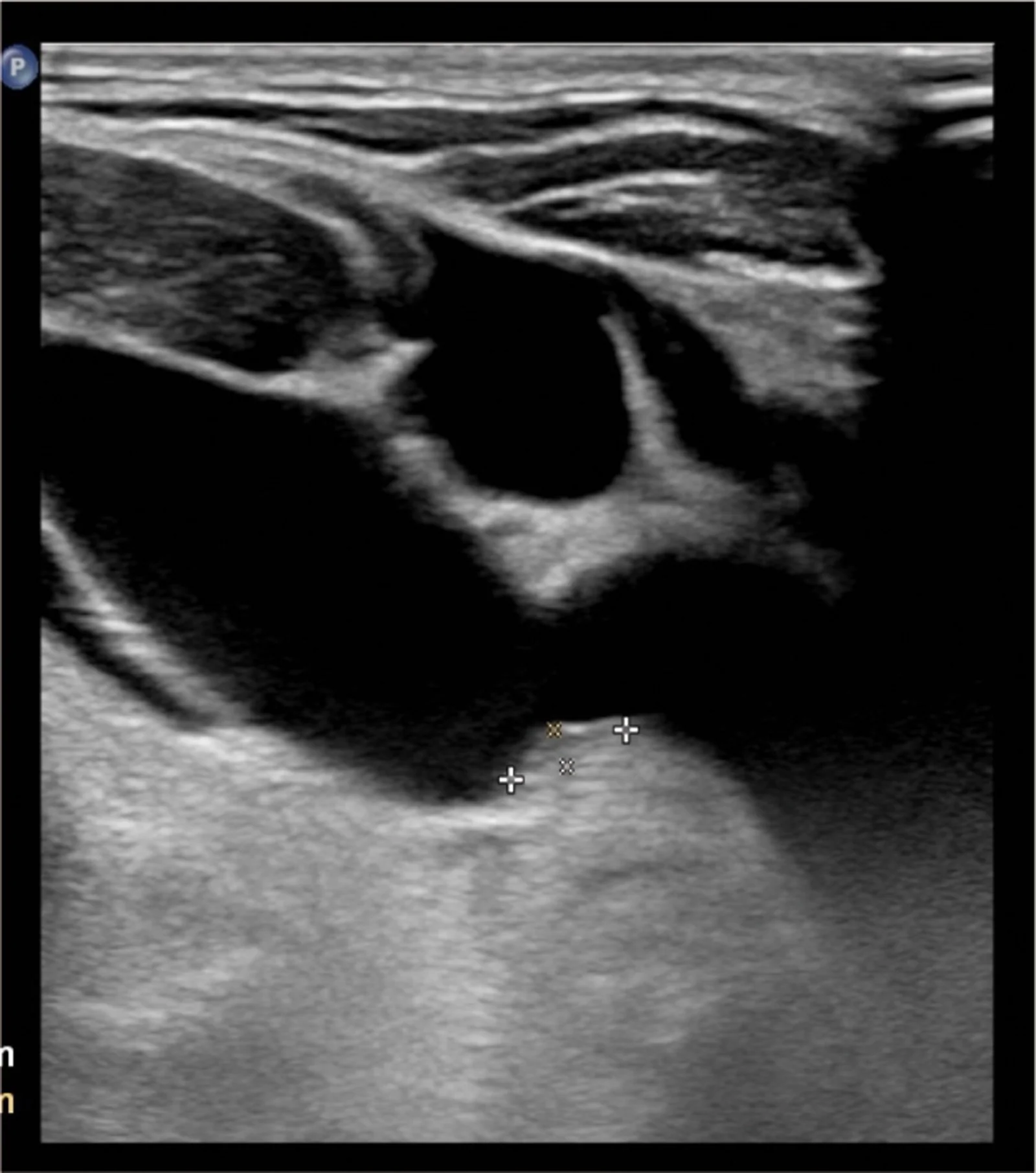
Vascular ultrasound. The cross indicates the atheromatous plaque, which measured 4.8 × 1.4 mm at the time of examination.

Management was shifted from a standard LDL-lowering strategy to a phytosterol-targeted intervention. This involved strict restriction of both animal fats and plant-based oils to eliminate the primary source of exogenous sterols. Pharmacological management was optimized by continuing ezetimibe (10 mg/day) and adding cholestyramine (1–2 g/day) to enhance the biliary excretion of accumulated sterols. Following intervention, the patient’s biochemical profile improved rapidly, with blood sitosterol levels decreasing to 6.64 mg/dL at 14 months. Clinically, the tuberous xanthomas on the Achilles tendons and patellar ligaments regressed significantly, and the buttock papules disappeared completely (*Figure 2*). Most notably, a follow-up vascular ultrasound at 14 months demonstrated satisfactory regression of the atheromatous plaque, which reduced in size from 12.4 × 2.7 to 4.8 × 1.4 mm, representing a 51.7% reduction in maximal thickness (*Figure 3*). The proportion of stomatocytes normalized to 30.4%, and the patient remained asymptomatic with no recurrence of joint pain. Subsequent follow-up has been scheduled to monitor sterol levels and long-term vascular stability.

## Discussion

While global prevalence was previously estimated at 1/200,000, recent screening of the 443 patients with suspected phenotypic FH identified eight patients (1.8%) who were homozygous or compound heterozygous for ABCG5/ABCG8 gene variants.^7^ The 7-year diagnostic odyssey of our patient—marked by misdiagnoses of fibrous histiocytoma and progressive pseudorheumatoid dysplasia—highlights a profound lack of familiarity with rare dyslipidaemias among clinicians.

Familial hypercholesterolaemia is an autosomal dominant disorder caused by pathogenic variants in LDLR, APOB, or PCSK9, diagnosed clinically based on LDL-C levels, physical findings (tendon xanthomas or corneal arcus), and family history of hypercholesterolaemia or premature arteriosclerotic cardiovascular disease.^8^ This patient had elevated LDL-C and xanthomas but no family history, precluding FH. In contrast, sitosterolaemia is autosomal recessive, often lacks a family history of hypercholesterolaemia, and may present with atypical features including haemolytic anaemia or stomatocytosis.^9^ In a cohort of 26 sitosterolaemia patients, xanthomas or hypercholesterolaemia were the most common presentations.^6^ Thus, sitosterolaemia should be a primary differential in patients with xanthomas, hypercholesterolaemia, or unexplained haematologic abnormalities without an FH family history.

A pivotal observation was the paradoxical progression of the carotid plaque (2.5 to 2.9 mm) between August 2022 and January 2023, despite significant LDL-C reduction. Under the presumptive diagnosis of FH, the patient was prescribed an animal-fat-restricted diet, which inadvertently increased plant oil consumptio.^10^ In individuals with ABCG5/G8 mutations, intestinal sterol absorption reaches 50%–80%, compared with <5% in healthy individuals.^11^ Consequently, a ‘heart-healthy’ diet for FH elevates atherogenic phytosterols, accelerating atherosclerosis. This ‘plant oil trap’ underscores the importance of early phytosterol measurement in the diagnostic evaluation of atypical hypercholesterolemia. This mechanistic concern is supported by epidemiological data: carotid intima-media thickness (IMT) abnormalities occur in 23%–25% of paediatric sitosterolaemia patients,^6,12^ significantly exceeding the 7.14% reported in age-matched FH patients,^8^ challenging the traditional view that phytosterols pose a lower vascular risk than LDL-C.

The most striking finding in this follow-up is the significant regression of the carotid plaque (from 2.7 to 1.4 mm) following the transition to a plant-oil-restricted diet. According to current clinical guidelines, patients with established atherosclerotic plaques are classified as very-high-risk, with a recommended LDL-C target of <55 mg/dL (1.4 mmol/L) to prevent major adverse cardiovascular events.^13^ Notably, this regression occurred only after blood sitosterol levels were suppressed below the threshold of 200 μmol/L (∼8.29 mg/dL), regardless of LDL-C fluctuations. This suggests that in sitosterolaemia, there may be a ‘threshold effect’ where intensive lowering of plant sterols is mandatory to achieve morphological reversal of atherosclerosis, regardless of LDL-C levels.

The long-term management of this patient focuses on strict dietary restriction of phytosterols combined with ezetimibe and cholestyramine as dual pharmacotherapy, with annual surveillance of plasma phytosterols, lipid profiles, haematologic parameters, xanthoma assessment, and cardiovascular imaging. Importantly, the management of such ‘phytosterolic plaques’ lacks a Class I recommendation in current paediatric protocols. The successful plaque regression achieved through plant-oil restriction and cholestyramine underscores the urgent need to establish specific therapeutic targets for plant sterols—parallel to LDL-C targets—in this population. To our knowledge, this is the first report to quantitatively document that phytosterolic plaques are highly dynamic and reversible in early childhood through targeted sterol-lowering therapy.

Early diagnosis is the cornerstone of preventing premature cardiovascular events in sitosterolaemia. Plasma phytosterol measurements should be considered early in young patients with atypical hypercholesterolaemia to exclude alternative diagnoses. Once identified, rigorous dietary and pharmacological intervention can reverse both cutaneous xanthomas and early-onset vascular damage in childhood. Improving clinician awareness and expanding access to phytosterol quantification are essential to mitigate the long-term cardiovascular burden of this underestimated disease.

## Supplementary Material

ytag603_Supplementary_Data

## Data Availability

The data underlying this study are available in this article. Any additional readers’ enquiries relating to this article will be shared upon reasonable request with the corresponding author.

## Lead author biography

Chao-chun Zou is a paediatric endocrinologist and geneticist, serving as a Chief Physician and Professor in the Department of Endocrinology at the Children's Hospital, Zhejiang University School of Medicine. He has profound expertise in paediatric growth-related endocrine and genetic disorders.

## Notes

**Consent:** The authors confirm that written consent for submitting and publishing this case report, including images and associated text, has been obtained from the patient in line with the COPE guidance.
