# Supplementary material for: Beyond cholesterol: regression of carotid plaques in an adolescent-a case report
Source: Eur Heart J Case Rep. 2026 Aug 18;10(9):ytag603. doi: 10.1093/ehjcr/ytag603 (PMC13541107; doi:10.1093/ehjcr/ytag603)
Supplement: Supplementary file 1 [file ytag603_supplementary_data.zip › supplemental files.docx]

Supplemental Figure 1. Pathology of elbow nodules and subcutaneous buttock papillae. (A) Pathology of elbow subcutaneous nodules showing multinucleated giant cell-forming neoplastic lesions with spindle-shaped and epithelioid differentiation (HE stain, 400 ×); (B) Pathology of buttock papules showing diffuse foamy histiocyte infiltration in the superficial and deep dermis (HE stain, 400 ×).

Supplemental Figure 2. Magnetic resonance imaging of Achilles tendon xanthoma. (A) Sagittal T1-weighted image showed uniform, spindle-shaped tendon thickening. (B) Sagittal T1-weighted fat-saturated image showed tendon thickening with signal intensity similar to that of adjacent muscle.
